# Male proboscis monkey cranionasal size and shape is associated with visual and acoustic signalling

**DOI:** 10.1038/s41598-024-60665-8

**Published:** 2024-05-23

**Authors:** Katharine L. Balolia, Pippa L. Fitzgerald

**Affiliations:** School of Archaeology and Anthropology, Australian National University, Canberra, 2601 Australia

**Keywords:** *Nasalis larvatus*, Primate, Geometric morphometrics, Cranium, Evolution, Sexual selection, Biological anthropology

## Abstract

The large nose adorned by adult male proboscis monkeys is hypothesised to serve as an audiovisual signal of sexual selection. It serves as a visual signal of male quality and social status, and as an acoustic signal, through the expression of loud, low-formant nasalised calls in dense rainforests, where visibility is poor. However, it is unclear how the male proboscis monkey nasal complex, including the internal structure of the nose, plays a role in visual or acoustic signalling. Here, we use cranionasal data to assess whether large noses found in male proboscis monkeys serve visual and/or acoustic signalling functions. Our findings support a visual signalling function for male nasal enlargement through a relatively high degree of nasal aperture sexual size dimorphism, the craniofacial region to which nasal soft tissue attaches. We additionally find nasal aperture size increases beyond dental maturity among male proboscis monkeys, consistent with the visual signalling hypothesis. We show that the cranionasal region has an acoustic signalling role through pronounced nasal cavity sexual shape dimorphism, wherein male nasal cavity shape allows the expression of loud, low-formant nasalised calls. Our findings provide robust support for the male proboscis monkey nasal complex serving both visual and acoustic functions.

## Introduction

Among many mammals, exaggerated sexually-selected facial traits in males serve as honest signals of fighting ability, and influence female mate choice^1–3^. Among primates, the large, bulbous nose of male adult proboscis monkeys (*Nasalis larvatus*) is unique, and has long been recognised as a sexually dimorphic trait, whose emergence has been linked to sexual selection through audiovisual signalling^3–11^. It serves as a visual signal, where male nasal enlargement is associated with the number of adult females per proboscis monkey breeding group, and the noses of males who are in breeding groups are significantly larger than those of males in non-breeding, all male groups^10^. There is evidence that enlarged nasal structures among male proboscis monkeys also play a role in acoustic communication, where larger formant ratios (a measure of vocal resonance properties) are tentatively associated with larger male nose size^10^. Therefore, enlarged nasal structures in male proboscis monkeys may allow them to emit louder or deeper nasal vocalisations, namely brays, honks and nasal roars, which communicate dominance and aggression, often in the context of intermale competition, intergroup communication and group protection^12–15^. This allows male proboscis monkeys with enlarged nasal structures to enhance their reproductive success.

The extent to which enlarged nasal structures in male proboscis monkeys evolved due to selective pressures associated with visual signalling or acoustic signalling, respectively, is difficult to ascertain by examining the nasal fleshy soft tissue alone^10^. However, understanding variation associated with male proboscis monkey cranionasal morphology, i.e. the nasal cavity (the vaulted chamber representing the bony cavity and the nasopharynx of the most superior and anterior part of the respiratory tract)^16,17^, including the nasal aperture (the bony anterior limit of the nasal cavity)^16^ may provide important insights into how and why enlarged nasal structures evolved in male proboscis monkeys, in association with audiovisual signalling. In the context of visual signalling, the nasal aperture is the bony region to which nasal soft tissue attaches^18^. This cranial attachment area therefore serves as a bony correlate of the nasal soft tissue morphology exhibited by male and female proboscis monkeys. Turning to acoustic signalling, male proboscis monkeys elicit two types of vocalisations that heavily rely on the nasal region of the vocal tract (herein referred to as ‘nasalised vocalisations’): honks and nasal roars, that are rarely elicited by females, if at all^6,12,14,19^. Male proboscis monkeys also elicit brays, a third type of nasalised vocalisation^10^, more frequently than do females^15^. Honks and nasal roars are characterised as loud calls based on their low fundamental frequencies (honks: 140 Hz; nasal roars: 137 Hz), and the high concentration of energy with which they are emitted^14^. Brays have fundamental frequencies of 85 Hz and are also characterised as loud calls^10,14^. By contrast, shrieks, which are the main vocalisation type expressed by females and immature proboscis monkeys^15^, are scream-like, higher fundamental frequency vocalisations (1350 Hz), are not considered to be loud calls, and do not heavily rely on the nasal region of the vocal tract^14,20^. As brays, honks and nasal roars are emitted by adult male proboscis monkeys using the nasal vocal tract^6,10,12,14,15,19^, if enlarged nasal structures are associated with acoustic communication, selection for increased nasal cavity size is expected among male proboscis monkeys, relative to females and other cercopithecoid taxa who do not elicit high amplitude nasalised loud calls. Further, the nasal cavity among male proboscis monkeys is expected to show a unique shape, capable of emitting loud and deep (high-amplitude, low formant) nasalised vocalisations.

Morphological traits that are under sexual selection associated with courtship and aggressive displays are expected to show positive static allometry because relatively large traits are an honest signal of an individual’s ability to win competitive encounters, and occur when the fitness gains of possessing a given trait are greater among larger animals than among smaller animals^21,22^. Among proboscis monkeys, if the nasal aperture or nasal cavity serves as a visual or acoustic signal of body size or fertility (i.e. through testis size)^10^, positive allometry in nasal aperture or cavity size is expected, depending on the function of nasal enlargement as an audiovisual trait. Sexual selection can additionally be detected using cranial variables based on assessments of the timing of maturity of the morphological trait under scrutiny, in the context of the timing of social maturity of the species being considered. For example, sagittal crest size among gorilla males, as measured using head profiles using digital photogrammetric data, is positively associated with the number of adult females per adult male, higher male offspring siring rates and offspring survival rates^23,24^. Male sagittal crest size, in conjunction with back breadth, is also associated with dominance rank, alpha male tenure length and the frequency of aggression^25^. Consistent with these behavioural data, the male gorilla sagittal crest shows positive static allometry when scaled against cranial size, and the timing of sagittal crest emergence among gorilla males in early adulthood coincides with the timing of social maturity^26^. Among proboscis monkeys, only adult males with large, developed noses live in uni-male, multi-female breeding groups, though younger males with smaller noses live exclusively in all-male groups^5,10,27,28^. These findings are consistent with evidence that male proboscis monkey large bulbous noses are a sexually selected trait, emerging in adulthood, and where older adult males have larger noses than their younger counterparts^5,10,28,29^. Therefore, if the male proboscis monkey nasal aperture or nasal cavity is associated with visual or acoustic signalling, size increases beyond dental maturity are expected.

As noted earlier, previous research has investigated soft tissue nasal morphology in the context of sexual selection through examining the relationships between relative nose size and body mass, testis size, number of adult females per breeding group, and vocal resonance properties^10^. However, no research has yet investigated enlarged nasal structures in male proboscis monkeys in the context of visual and acoustic signalling, using cranionasal data. In this study, we evaluate whether there is support for the ‘visual signalling hypothesis’ (the hypothesis that enlarged nasal structures evolved as a visual signal of male quality) and the ‘acoustic signalling hypothesis’ (the hypothesis that enlarged nasal structures evolved to allow males to emit enhanced nasalised vocalisations) in male proboscis monkeys. We investigate whether proboscis monkey sexual size dimorphism of the nasal aperture, and sexual size and shape dimorphism of the nasal cavity, exceeds that of three other cercopithecoid species (*Cercopithecus mitis, Colobus polykomos* and *Macaca fascicularis*). We assess whether proboscis monkey cranionasal morphology is larger than expected for a given cranial size by examining the allometric relationships between nasal aperture size, nasal cavity size and cranial size among male and female proboscis monkeys. Finally, we assess nasal aperture and nasal cavity size changes with age beyond dental maturity. We outline our predictions under the ‘visual signalling hypothesis’ and the ‘acoustic signalling hypothesis’ for each analysis in Table 1. The visual signalling hypothesis and the acoustic signalling hypothesis are not mutually exclusive, as large noses among male proboscis monkeys could simultaneously function as both a visual and as an acoustic signal.

**Table 1:** Predictions under the ‘visual signalling hypothesis’ and the ‘acoustic signalling hypothesis’ for enlarged nasal structures in male proboscis monkeys, and support for each hypothesis in the present study.

Analysis	Visual signalling hypothesis predictions	Acoustic signalling hypothesis predictions	Present study findings
Sexual size dimorphism	Proboscis monkey nasal aperture size dimorphism will exceed nasal cavity size dimorphism. Proboscis monkeys will show increased nasal aperture size dimorphism, relative to other cercopithecoid species, driven by increased nasal aperture size in male proboscis monkeys.	No predictions are made under this hypothesis.	Findings of increased nasal aperture size dimorphism, relative to nasal cavity size dimorphism in proboscis monkeys, **support the visual signalling hypothesis**. Findings of increased nasal aperture size dimorphism in proboscis monkeys, relative to three other cercopithecoid species, also **support the visual signalling hypothesis**.
Sexual shape dimorphism	No predictions are made under this hypothesis.	Proboscis monkeys will show increased nasal cavity shape dimorphism, relative to other cercopithecoid species, who do not use nasalised vocalisations for auditory communication. Selection for increased sexual shape dimorphism in proboscis monkeys is expected, to enhance the capacity for male proboscis monkeys to emit low formant, loud nasalised vocalisations.	Findings of increased nasal cavity shape dimorphism in proboscis monkeys, relative to three other cercopithecoid species,** support the acoustic signalling** **hypothesis**. Visualisations of proboscis monkey nasal cavity sexual shape dimorphism are consistent with the ability to emit low formant, loud nasalised vocalisations, **consistent with the acoustic signalling hypothesis**. See the discussion section for further details.
Allometry	There will be a positive allometric relationship (slope > 1) between nasal aperture size and cranial size in male proboscis monkeys, but not in female proboscis monkeys. There will be an isometric relationship (slope = 1) between proboscis monkey nasal cavity size and cranial size in both sexes.	There will be a positive allometric relationship (slope > 1) between nasal cavity size and cranial size in male proboscis monkeys, but not in female proboscis monkeys.	**Neither the visual signalling hypothesis, nor the acoustic signalling hypothesis are supported**. However, the lack of a significant relationship between nasal aperture size and cranial size (i.e. the finding that nasal aperture size variation is independent of cranial size variation) may be indicative of selection on nasal aperture size in proboscis monkeys, showing **tentative support for the visual signalling hypothesis**. See the discussion section for further details.
Growth beyond dental maturity	There will be an increase in male proboscis monkey nasal aperture size beyond dental maturity, associated with size increases of the fleshy soft tissue with increasing adulthood age. No associated size increases are expected in female proboscis monkeys, or for nasal cavity size in either sex. These size increases are expected, independent of increases in cranial size.	There will be an increase in male proboscis monkey nasal cavity size beyond dental maturity, associated with loud, low formant nasalised vocalisations with increasing adulthood age. No associated size increases are expected in female proboscis monkeys. These size increases are expected, independent of increases in cranial size.	Findings of nasal aperture growth beyond dental maturity among proboscis monkey males, in the absence of cranial growth, though no similar relationship is observed among female proboscis monkeys **support the visual signalling hypothesis**. Findings of no growth beyond dental maturity for the nasal cavity shows that **the acoustic signalling hypothesis is not supported**.

## Results

### Sexual size dimorphism

The nasal aperture of male proboscis monkeys is 29% larger than that of females (Table 2). This relationship remains significant after controlling for body mass (t_(31)_ = 2.550, p = 0.016). The degree of nasal aperture sexual size dimorphism in proboscis monkeys exceeds that of the other three cercopithecoid species, where among these species, male nasal aperture size is 7–15% larger than that of females (Table 2).

**Table 2:** Nasal aperture and nasal cavity sexual size dimorphism in *Nasalis larvatus*, *Colobus polykomos*, *Cercopithecus mitis* and *Macaca fascicularis*.

Species	Males	Females	Nasal aperture sexual size dimorphism				Nasal cavity sexual size dimorphism			
			Male mean (mm)	Female mean (mm)	ISD	*t*-test	Male mean (mm)	Female mean (mm)	ISD	*t*-test
*Nasalis larvatus*	19/18	14/14	17.03	13.21	1.29	*t*_(27.287)_ = 10.498, p < 0.001	23.27	18.52	1.26	*t*_(30)_ = 13.722, p < 0.001
*Colobus polykomos*	20/20	20/20	15.10	14.17	1.07	*t*_(32.933)_ = 3.224, p = 0.003	19.25	17.94	1.07	*t*_(32.347)_ = 5.277, p < 0.001
*Cercopithecus mitis*	18/18	12/12	15.08	13.30	1.13	*t*_(28)_ = 4.191, p < 0.001	20.36	17.64	1.15	*t*_(28)_ = 5.548, p < 0.001
*Macaca fascicularis*	20/20	19/19	13.92	12.13	1.15	*t*_(37)_ = 5.416, p < 0.001	20.47	17.43	1.17	*t*_(37)_ = 6.895, p < 0.001

*ISD* Index of Sexual Dimorphism. The number of male and female specimens used in each analysis is denoted by the number of specimens used in nasal aperture size analyses/the number of specimens used in nasal cavity size analyses.

The nasal cavity of male proboscis monkeys is 26% larger than that of females (Table 2). This relationship also remains significant after correcting for body size (t_(30)_ = 3.137, p = 0.004). Similar to what is observed for the nasal aperture, nasal cavity sexual size dimorphism in proboscis monkeys exceeds that of the other three cercopithecoid species, where among these species, male nasal cavity size is 7–17% larger than that of females (Table 2).

### Sexual shape dimorphism

Proboscis monkeys show increased nasal cavity sexual shape dimorphism, relative to the three other cercopithecoid taxa (Table 3). Despite proboscis monkeys showing higher levels of body mass dimorphism than blue monkeys (*C. mitis*) and crab-eating macaques (*M. fascicularis*)^30,31^ (Supplementary Table 1), the percentage of nasal cavity shape variation accounted for by size in proboscis monkeys is lower than that of these two species (Table 3). This indicates that the high level of nasal cavity sexual shape dimorphism found in proboscis monkeys is not an artifact of size.

**Table 3:** Nasal cavity sexual shape dimorphism and nasal cavity shape variation explained by size in *Nasalis larvatus*, *Colobus polykomos*, *Cercopithecus mitis* and *Macaca fascicularis*.

Species	Males	Females	Nasal cavity male–female PD	Nasal cavity shape variation explained by size
*Nasalis larvatus*	18	13	0.0684, p < 0.0001	16.13%, p < 0.0001
*Colobus polykomos*	20	20	0.0247, p = 0.054, ns	4.69%, p = 0.035
*Cercopithecus mitis*	18	12	0.0442, p = 0.0001	18.94%, p < 0.0001
*Macaca fascicularis*	20	19	0.0308, p = 0.0002	21.04%, p < 0.0001

*PD* Procrustes Distance, *ns* not significant.

**Figure 1:**
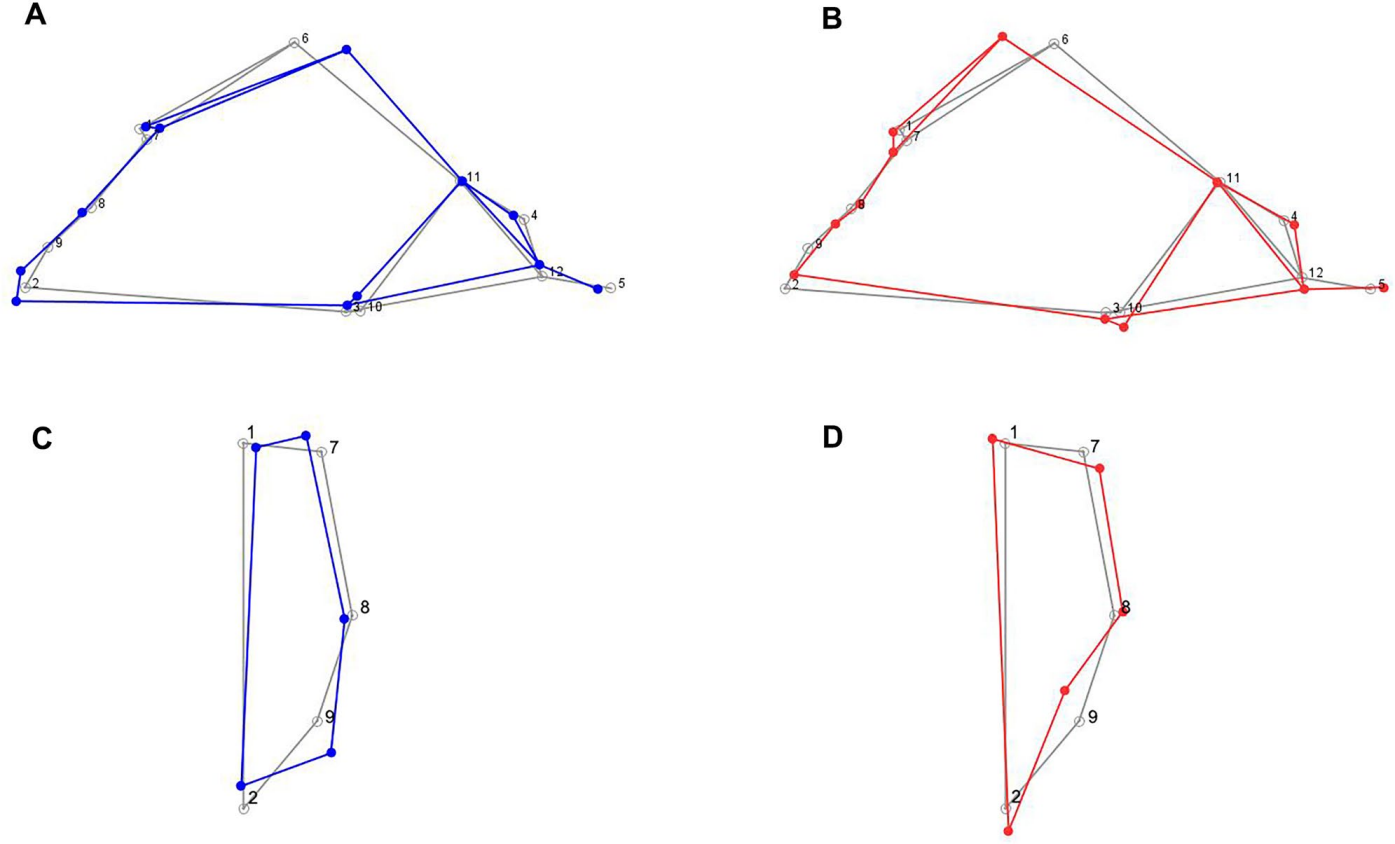
Wireframes showing mean nasal cavity shapes (Plots **A**,**B**) and mean nasal aperture shapes (Plots **C**,**D**) in proboscis monkeys. Plot (**A**) = mean male nasal cavity shape (blue), Plot (**B**) = mean female nasal cavity shape (red), Plot (**C**) = mean male nasal aperture shape (blue), Plot (**D**) = mean female nasal aperture shape (red). The wireframes shown in grey represent the mean nasal cavity and aperture shapes for males and females combined. Sex-specific nasal cavity and nasal aperture shapes have been scaled to a factor of 5 to more easily visualise which landmarks contribute to shape variation. Nasal cavity and nasal aperture landmarks are depicted in Supplementary Fig. S1, and landmark definitions are provided in Supplementary Table S2.

Visualisations of proboscis monkeys male and female nasal cavity shapes, relative to the mean proboscis monkey nasal cavity shape, show that there are sex differences in the superior and inferior nasal margins, and in the relative position of the superior lacrimal fossa (Fig. 1). Shape comparisons of the superior and inferior nasal margins show that the lateral aspect of the superior nasal margin among male proboscis monkeys (LM7) is more superiorly placed, relative to females, and the lateral aspect of the inferior nasal margin among males (LM9) is more inferiorly and laterally placed, relative to females (Fig. 1). However, the relative width of the nose at the widest point (LM8) is similar in both sexes, with females displaying a relatively wide superior nasal aperture (Fig. 1). Therefore, there are sex differences in the relative shape of the nasal aperture opening, where the nasal aperture is more ‘eggplant’ shaped in males, whilst resembling an ‘upside down pear’ in females (Fig. 1). The superior lacrimal fossa (LM6) is posteriorly positioned in males, relative to females, resulting in a relatively longer and lower nasal chamber in lateral aspect among males (Fig. 1). There are no substantial sex differences in the relative position of rhinion, nasospinale and alare; nor are there substantial differences between males and females in the posterior landmarks of the nasal cavity (Fig. 1). We consider how these nasal cavity shape differences are associated with acoustic signalling in the discussion section.

### Allometry

**Figure 2:**
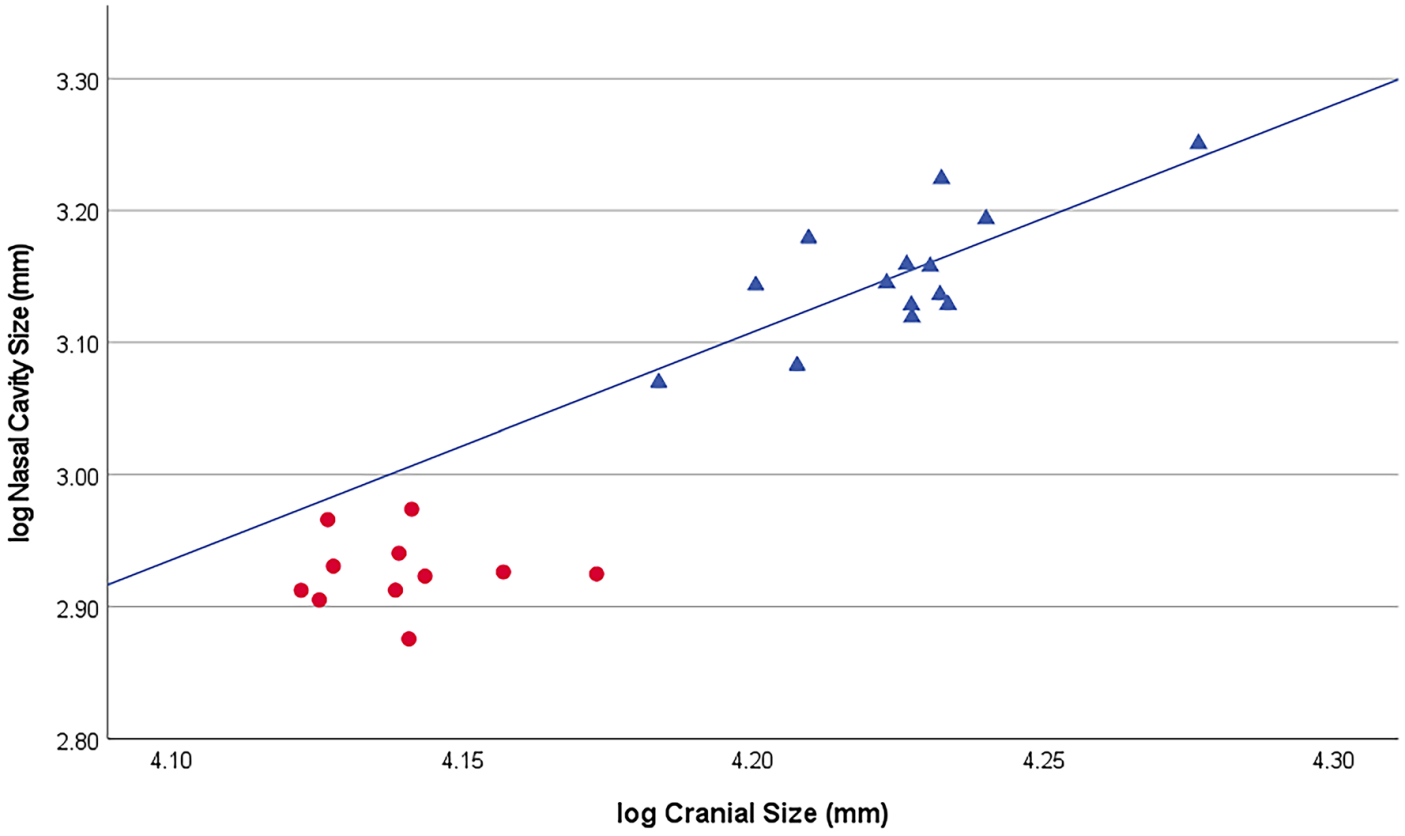
Log cranial size (x axis) vs log nasal cavity size (y axis) for proboscis monkeys. The blue triangles denote males and the red circles denote females. The male slope is statistically significant (F_(1,12)_ = 15.244, p = 0.002). No female regression line is shown as there is no significant relationship between log cranial size and log nasal cavity size among females (F_(1,9)_ = 0.001, p = 0.971).

There is no significant relationship between log nasal aperture size and log cranial size among male or female proboscis monkeys (males: F_(1,13)_ = 1.855, p = 0.196; females: F_(1,9)_ = 0.128, p = 0.728), wherein among both males and females, nasal aperture size varies independently of cranial size. By contrast, the relationship between log nasal cavity size and log cranial size among male proboscis monkeys is significant (F_(1,12)_ = 15.244, p = 0.002; Pearson’s *r* = 0.748, n = 14, p = 0.002; Fig. 2), wherein 52% of the variation in male nasal cavity size can be explained by cranial size. This relationship does not deviate from isometry (y = − 4.126 + 1.72x; 95% confidence interval: 0.761 < *B* < 2.683). The relationship between log nasal cavity size and log cranial size among female proboscis monkeys is not statistically significant (F_(1,9)_ = 0.001, p = 0.971).

### Growth beyond dental maturity

Male proboscis monkeys show a positive, significant relationship between nasal aperture size and relative age (Spearman’s *r* = 0.453, n = 19, p = 0.026). No significant relationship between these two variables is found among female proboscis monkeys (Spearman’s *r* = 0.191, n = 14, p = 0.256). There is no significant relationship between nasal cavity size and relative age in male or female proboscis monkeys (males: Spearman’s *r* = 0.331, n = 18, p = 0.09; females: Spearman’s* r* = 0.007, n = 14, p = 0.491), nor is there a significant relationship between cranial size and relative age in either sex (males: Spearman’s *r* = 0.364, n = 15, p = 0.091; females: Spearman’s *r* = − 0.132, n = 13, p = 0.334).

## Discussion

Our findings support both the visual and acoustic signalling hypotheses for enlarged nasal structures among male proboscis monkeys (Table 1). The visual signalling hypothesis is supported based on large nasal aperture size among males, i.e. the bony attachment area of the nasal soft tissue^18^, and male nasal aperture growth beyond dental maturity, consistent with enlarged nasal structures developing in mid-adulthood among some male proboscis monkeys^5,10,27,28^. The acoustic signalling hypothesis is supported based on increased nasal cavity sexual shape dimorphism among proboscis monkeys, and a longer, lower nasal cavity shape among males. Male proboscis monkey nasal cavity shape and enlarged nasal cavity size is consistent with the expression of low formant nasalised vocalisations. These include brays, honks and nasal roars, which are emitted at low fundamental frequencies (85–140 Hz) with a high concentration of energy, consistent with their categorisations as loud calls^14^. In particular, based on evidence that nasal and paranasal cavity morphology is associated with variation in formant frequency, in conjunction with overall vocal tract morphology^32^, the low, long and large nasal cavity observed in male proboscis monkeys found as part of the present study may facilitate the expression of the loud, low formant nasalised vocalisations observed among male proboscis monkeys^6,12,14,15,19^. High intensity primate vocalisations with low fundamental frequencies are associated with the condition of the individual and dominance status^33,34^ and therefore may be an honest indication of fighting ability, with lower fundamental frequency vocalisations preferred by females^34^. Our study findings further show sexual shape differences in the proboscis monkey nasal aperture, with an ‘eggplant’ shape in males, and an ‘upside down pear’ shape in females. Male nasal aperture shape therefore allows for greater concentration of sound to be passed through the nasal aperture, potentially allowing for high intensity sounds with low formants (increased resonance) to be emitted, consistent with the source-filter theory of vocalisations in non-human mammals as honest signals of male quality, body size or age^35,36^. The low formant frequency and the high amplitude of male proboscis monkey nasalised loud calls may be particularly important given that they live in forested environments, where the sounds expressed by animals need to propagate sufficiently to communicate information to individuals with whom they are not in visual proximity^37,38^.

Contrary to our expectations, in the present study we find no association between nasal aperture size and cranial size in either sex, though an isometric relationship between nasal cavity size and cranial size is observed among males. While our findings do not support either the visual signalling hypothesis or the acoustic signalling hypothesis under our study predictions, it is noteworthy that among male proboscis monkeys, nasal aperture size does not show a significant relationship with cranial size, while nasal cavity size does show this association. This indicates that nasal aperture size in male proboscis monkeys varies independently of nasal cavity size, consistent with the visual signalling hypothesis. Under this scenario, nasal aperture size variation is associated with the size of the fleshy nose and the timing of its enlargement, independent of cranial size. This interpretation is consistent with reports that nasal enhancement may be delayed in male proboscis monkeys until they have an opportunity to take over a breeding group^29^. There have been similar reports of the delayed adulthood onset of soft tissue secondary sexual characteristics among orangutans and capuchin monkeys^39,40^. Under this scenario, we suggest that if male proboscis monkey noses only increase when an opportunity to take over a breeding group presents itself, i.e. at different stages of adulthood based on when the opportunity arises, an association between nasal aperture size with cranial size may not expected, consistent with our study findings.

In the present study, we show that male proboscis monkey cranionasal morphology is associated with sexual selection, through both visual and acoustic signalling. The fleshy soft tissue of the nasal apparatus has previously been associated with audiovisual signalling among male proboscis monkeys^10^ and it is therefore likely that internal nasal cavity size and shape work in conjunction with the enhanced fleshy nose tissue to emit the unique nasalised vocalisations found in this species. This is in addition to the role of the nasal soft tissue, and its bony attachment area correlates, in visual signalling. Behavioural data support the interpretation that the nasal soft tissue and nasal cavity work together to emit unique proboscis monkey nasalised vocalisations, where during nasal honks, male proboscis monkeys reportedly rigidly straighten their noses in an upwards direction with each honk^19^. A similar phenomenon has been observed in male saigas, who too have prominent nasal morphology, and who tense and extend their noses when emitting nasal roars^41^. Therefore, enlarged nasal morphology in the context of audiovisual signalling among proboscis monkeys, while unique among primates, has been observed in other mammals. The results of our study build on previous evidence that primate craniofacial regions can carry a signal of sexual selection^26,42,43^ and our findings have implications in their potential applications for interpreting extinct primate social behaviour.

## Methods

### Study sample and 3D model acquisition

The sample consists of 142 dentally mature cranial specimens belonging to four cercopithecoid species (*C. mitis, C. polykomos, M. fasciluaris, N. larvatus*) (Supplementary Table S1). The species of interest is *N. larvatus*, and we selected the three other cercopithecoid species as a comparative sample to compare *N. larvatus* cranionasal sexual size and shape dimorphism. We selected *C. polykomos* as a second colobine species that is closely related to *N. larvatus*, and selected *M. fascicularis* and *C. mitis* as more distantly related cercopithecoid species that show moderate-high levels of body mass dimorphism, approaching the level observed in *N. larvatus*^31^ (Supplementary Table S1). None of the three comparative taxa (*C. mitis, C. polykomos, M. fasciluaris*) use nasalised loud calls as part of their modes of auditory communication. 3D models were obtained using a Polyga HDI Advance RX4 or a LMI Technologies HDI 3D scanner. Differences in the type of scanner used are unlikely to introduce substantial measurement error based on the specimen size of the species sampled as part of this study^44^.

### Morphometric size and shape variables

We quantified nasal aperture and nasal cavity size and shape using 3D landmarks, adapted from Noback et al.^17^, taken from 3D surface models using Checkpoint v. 2022.12.16.0419 (Supplementary Fig. S1; Supplementary Table S2). We quantified nasal aperture size as the geometric mean of three linear measurements, and quantified nasal cavity size as the geometric mean of seven linear measurements (Supplementary Table S3).

We estimated body mass as follows: orbital breadth (the minimum distance between maxillofrontale and ectoconchion) × orbital height (the minimum distance between upper and lower margins of the orbital cavity, taken at a right angle to orbital breadth) × π, based on evidence that orbital area as an ellipse is a strong predictor of body mass^45^. We quantified cranial size as the geometric mean of the following three measurements: (1) cranial height: the distance between superior cranial vault (the point the sagittal plane of the cranial vault that intersects with the posterior limits of the zygomatic process in Frankfurt horizontal) and basion (the anterior-most point of the foramen magnum), (2) cranial length: the distance between glabella (the most anterior point between the orbits at the midline) and opisthocranion (the most posterior point of the cranium at the Frankfurt horizontal at the midline), and (3) cranial breadth: the distance between the left and right midpoints of the zygomatic arch at the widest point.

We quantified nasal cavity shape by performing generalised Procrustes analysis (GPA) on 12 3D landmarks (Supplementary Fig. S1; Supplementary Table S2) for all four species combined. 3D landmarks were scaled to a standard centroid size, then translated and rotated to minimise the squared distances between sets of landmarks^46^.

### Estimating relative adult age

To assess relative adulthood age in *N. larvatus* specimens, we ranked specimens based on the degree of upper molar wear^26,47^ using screenshots of the dentition of each specimen, taken from 3D surface models. The youngest adult specimens show relatively low amounts of molar wear and the oldest specimens show relatively heavy molar wear. Anterior dentition and premolar wear were taken into account when there was not enough molar wear to adequately rank specimens. Males and females were ranked separately.

### Statistical analysis

#### Sexual size dimorphism

We quantified nasal aperture and nasal cavity sexual size dimorphism by calculating the index of sexual size dimorphism (ISD), calculated as mean male size/mean female size^42^. We tested for statistically significant differences between mean male and mean female size values using Student’s *t*-tests. When performing *t*-tests, we used Levene’s test for homogeneity of variance. When the homogeneity of variance assumption was violated, we reported Welch’s test of equality of means. We performed sexual size dimorphism tests using SPSS v. 29.

#### Sexual shape dimorphism

We quantified nasal cavity sexual shape dimorphism by calculating the Procrustes Distance (PD) between the mean male nasal cavity shape and the mean female nasal cavity shape, using Procrustes shape coordinates from the GPA described earlier. We used permutation tests to assess whether the male-female PD was significant (10,000 permutations per analysis). We assessed the percentage of nasal cavity shape variation that was influenced by nasal cavity size using pooled within-group regression analyses and using permutation tests (10,000 permutations per analysis to assess statistical significance). We visualised *N. larvatus* nasal cavity shape using wireframes to compare the mean male, and mean female shape, respectively, with the mean *N. larvatus* shape. Shape visualisations were performed using Procrustes shape coordinates from GPAs, performed on the *N. larvatus* dataset. Mean male and mean female shape visualisations were performed using a scaling factor of five. We performed shape analyses and wireframe visualisations using MorphoJ v. 1.07a^48^.

#### Allometry

We assessed whether there were statistically significant relationships between nasal aperture size and nasal cavity size, respectively, and cranial size using ordinary least squared (OLS) regression on log transformed variables, using log cranial size as the dependent variable, and log nasal aperture or log nasal cavity size as the independent variable. We used ANOVA tests to assess model significance. When the model was significant we used Pearson’s correlation coefficient to assess the relationship between variables, and used the adjusted R^2^ value to assess how much nasal size variation is explained by cranial size variation. For models that were statistically significant, we tested for isometry by calculating the 95% confidence intervals of the regression value *B*, where *B* > 1 = positive allometry, *B* = 1 = isometry, and *B* < 1 = negative allometry.

#### Growth beyond dental maturity

We assessed whether there were nasal aperture, nasal cavity or cranial size increases with age beyond dental maturity using Spearman’s correlations between these size variables and tooth wear rank (1-tailed test).

### Supplementary Information

Supplementary Information.

## Data Availability

The dataset used in this study is available via Figshare: 10.6084/m9.figshare.24845043.
