# Supplementary material for: Male proboscis monkey cranionasal size and shape is associated with visual and acoustic signalling
Source: Sci Rep. 2024 May 23;14:10715. doi: 10.1038/s41598-024-60665-8 (PMC11116372; doi:10.1038/s41598-024-60665-8)
Supplement: Supplementary file 1 — Supplementary Information. [file 41598_2024_60665_MOESM1_ESM.pdf]

Supplementary Information

Male proboscis monkey cranionasal size and shape is associated with visual and acoustic signalling

Katharine L. Balolia, Pippa L. Fitzgerald

Supplementary Table S1. Study sample, including sex breakdown, specimen repositories and body mass index of sexual dimorphism (ISD).

Species	Males	Females	Repositories	Body mass ISD [1]
Cercopithecus mitis	18	12	Royal Museum of Central Africa, Tervuren; Smithsonian National Museum of Natural History, Washington DC	1.741
Colobus polykomos	20	20	Royal Museum of Central Africa, Tervuren; Smithsonian National Museum of Natural History, Washington DC	1.193
Macaca fascicularis	20	19	Field Museum of Natural History, Chicago; Smithsonian National Museum of Natural History, Washington DC	1.577
Nasalis larvatus	19	14	American Museum of Natural History, New York; Field Museum of Natural History, Chicago; Smithsonian National Museum of Natural History, Washington DC	2.077

[1] Plavcan, J. M. (2002). Taxonomic variation in the patterns of craniofacial dimorphism in primates. *Journal of Human Evolution, 42,* 579-608.

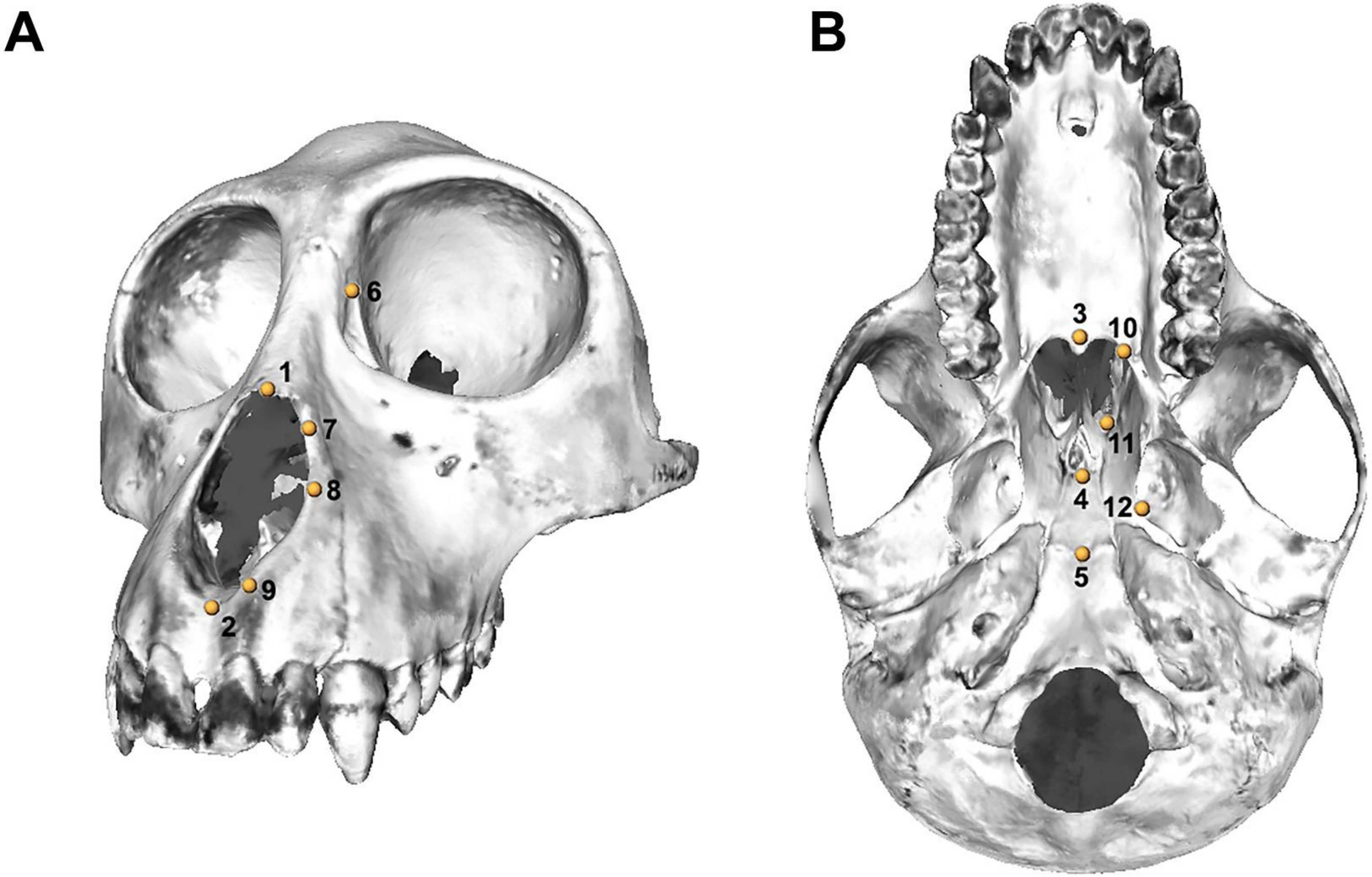

Supplementary Figure S1. Depiction of the 3D landmarks used to quantify the nasal cavity (landmarks 1-12), including the nasal aperture (landmarks 1, 2, 7, 8 & 9), applied to a female proboscis monkey specimen (AMNH103466, Morphosource Media ID 000355958). Plot A = angled frontal view; Plot B = inferior view. Landmark definitions are provided in Supplementary Table S2.

Supplementary Table S2. 3D Landmarks (LMs) used to quantify nasal cavity and nasal aperture morphology. Landmarks are depicted in Supplementary Figure S1.

LM#	Landmark name and definition	Nasal aperture	Nasal cavity
1	Rhinion: the point at the tip of the internasal suture at the midline	X	X
2	Nasospinale: the point on the lower rim of the nasal cavity at the midline	X	X
3	Staphylion: the point on the median palatine suture that crosses a line drawn from the deepest part of the arches of the horizontal plate of the palatine bones at the midline		X
4	Posterior vomer: Most posterior point on the vomer at the midline		X
5	Spheno-occipital synchondrosis: The meeting point between the basisphenoid and basioccipital at the midline		X
6	Superior lacrimal fossa: The most superior point on the lacrimal fossa (left)		X
7	Superior nasal margin: The most superior margin of the nasal aperture (left)	X	X
8	Alare: the most lateral point on the margin of the nasal aperture (left)	X	X
9	Inferior nasal margin: The point of inflection at the inferior margin of the nasal aperture (left)	X	X
10	Inferior choana: The most inferior and lateral margin of the choanal corner (left)		X
11	Superior choana: The most superior and posterior margin of the choana (left)		X
12	Posterior pterygoid plate: The most posterior and superior end of the medial pterygoid plate (left)		X

Supplementary Table S3. 2D measurements used to quantify nasal cavity and nasal aperture size. Landmarks (LMs) are depicted in Supplementary Figure S1.

Abbreviation	Definition	Nasal aperture	Nasal cavity
NASH	Nasal height: Rhinion (LM1) to Nasospinale (LM2)	X	X
UPNH	Upper nasal height: Rhinon (LM1) to Alare (LM8)	X	X
LONH	Lower nasal height: Alare (LM8) to Nasopinale (LM2)	X	X
RHLF	Rhinion (LM1) to Superior Lacrimal Fossa (LM6)		X
LFPV	Superior Lacrimal Fossa (LM6) to Posterior Vomer (LM4)		X
PVST	Posterior Vomer (LM4) to Staphylion (LM3)		X
STNA	Staphylion (LM3) to Nasospinale (LM2)		X
